# The health and social implications of household air pollution and respiratory diseases

**DOI:** 10.1038/s41533-019-0126-x

**Published:** 2019-04-26

**Authors:** Suzanne M. Simkovich, Dina Goodman, Christian Roa, Mary E. Crocker, Gonzalo E. Gianella, Bruce J. Kirenga, Robert A. Wise, William Checkley

**Affiliations:** 1 Division of Pulmonary and Critical Care, School of Medicine, Johns Hopkins University, Baltimore, MD USA; 2 Center for Global Non-Communicable Diseases, School of Medicine, Johns Hopkins University, Baltimore, MD USA; 3 Division of Pulmonary and Sleep Medicine, University of Washington, Seattle Children’s Hospital, Seattle, WA USA; 4 Facultad de Medicina Alberto Hurtado, Universidad Peruana Cayetano Heredia, Lima, Peru; 5 Servicio de Neumología, Unidad de Cuidados Intensivos, Clinica Ricardo Palma, Lima, Peru; 6 Makerere Lung Institute, Makerere University, Kampala, Uganda; 7 Pulmonology Unit, Department of Medicine, Makerere University, Mulago Hospital, Kampala, Uganda

**Keywords:** Respiratory tract diseases, Epidemiology, Epidemiology

## Abstract

Approximately three billion individuals are exposed to household air pollution (HAP) from the burning of biomass fuels worldwide. Household air pollution is responsible for 2.9 million annual deaths and causes significant health, economic and social consequences, particularly in low- and middle-income countries. Although there is biological plausibility to draw an association between HAP exposure and respiratory diseases, existing evidence is either lacking or conflicting. We abstracted systematic reviews and meta-analyses for summaries available for common respiratory diseases in any age group and performed a literature search to complement these reviews with newly published studies. Based on the literature summarized in this review, HAP exposure has been associated with acute respiratory infections, tuberculosis, asthma, chronic obstructive pulmonary disease, pneumoconiosis, head and neck cancers, and lung cancer. No study, however, has established a causal link between HAP exposure and respiratory disease. Furthermore, few studies have controlled for tobacco smoke exposure and outdoor air pollution. More studies with consistent diagnostic criteria and exposure monitoring are needed to accurately document the association between household air pollution exposure and respiratory disease. Better environmental exposure monitoring is critical to better separate the contributions of household air pollution from that of other exposures, including ambient air pollution and tobacco smoking. Clinicians should be aware that patients with current or past HAP exposure are at increased risk for respiratory diseases or malignancies and may want to consider earlier screening in this population.

## Introduction

Respiratory diseases are responsible for a significant burden worldwide from direct healthcare costs, significant disability, premature mortality, lost productivity and social consequences. Specifically, chronic respiratory diseases are estimated to result in 92.5 million disability-adjusted life years (DALYs) lost in 2016 worldwide.^1^ There is limited published data on the health expenditures for respiratory disease outside of the United States (US) and the European Union (EU).^2^ Furthermore, available statistics grossly underestimate health costs due to widespread underdiagnoses of respiratory disease.^2^ For the 28 countries in the EU, lung disease is estimated to cost €379.6 billion and results in an annual loss of 5.2 million DALYs, valued at an additional €300 billion.^2^ In the US, lung diseases cost an estimated $129 billion, with $106 billion of this attributed to chronic obstructive pulmonary disease (COPD), asthma, and pneumonia.^3^

Individuals in low- and middle-income countries (LMICs) have different exposures, and consequently risk factors, for the development of respiratory diseases as compared to those in higher income countries.^4^ Household air pollution (HAP) exposure is an important attributable risk factor for both acute and chronic respiratory diseases in LMICs, including acute respiratory infections,^5–8^ tuberculosis,^9,10^ asthma,^5^ COPD,^5,11^ pneumoconiosis,^12^ head and neck cancers,^13^ and lung cancer.^14,15^ HAP exposure results from the incomplete combustion of biomass fuels (e.g., wood, dung, agricultural crop waste, and coal) during cooking and heating. Almost three billion individuals, 42.2% of the world population, continue to cook with biomass fuels due to inadequate access to clean energy.^16^ According to the 2016 Global Burden of Disease estimates, HAP was responsible for 2.9 million annual deaths and 81.1 million DALYs lost.^1^ These estimates show that 26% of HAP deaths were attributed to lower respiratory infections, 5% to tracheal, bronchial and lung cancers, and 23% to COPD.^17^ Other respiratory diseases were not included in the 2016 study.^18^ Although HAP exposure affects all members of the household, women often have the highest risk of exposure due to their involvement in the cooking process.^4^ Children are also often close to their mothers and therefore can be exposed to HAP from a young age.^4^

Exposure to HAP not only has deleterious health effects, but also has important social consequences. Welfare and labor income losses are estimated at $1.6 trillion and $94 billion, respectively, due to lost productivity and poor health from HAP exposure.^18,19^ These losses are reflected in the poverty trap, a phenomenon where those who are in poor health, resulting from an environmental exposure such as HAP, cannot work or if they can work, their wages are lower. These individuals then cannot afford goods and services that would improve their health, feeding into the vicious cycle of poverty.^19^ Women and children are often burdened with biomass fuel collection,^20^ a time consuming and potentially dangerous task as many women are subjected to violence leaving their homes to collect the fuel.^4,19^ This obligation forces many children to miss school and women to have fewer opportunities to engage in other economic activities.^20^ Other health and environmental consequences of HAP include worse ambient air pollution, deforestation, and loss of habitat for wildlife.^21–24^

Despite the Global Burden Disease Estimates of HAP deaths and DALYs, the relationship between HAP and respiratory diseases remains poorly characterized. One potential concern is the need to appropriately attribute HAP exposure to respiratory diseases, which requires better environmental exposure monitoring and occupational exposure screening to separate HAP from the effects of tobacco smoking, air pollution or other occupational exposures. As cigarette smoking has a been attributed to negative respiratory health outcomes, such as pneumonia,^25^ asthma,^5^ tuberculosis (TB),^26^ and COPD,^27^ several studies have also sought to evaluate the association between HAP exposure and respiratory disease. For decades, researchers have been trying to characterize the effects of HAP exposure on the lungs, using the term “hut lung” to describe the negative consequences of HAP exposure on respiratory health.^28^ Recently, biological mechanisms have since been further elucidated, linking noxious chemicals and particles in HAP to inflammation. For example, particulate matter, one of the more commonly studied pollutants caused by incomplete biomass combustion, has been hypothesized to stimulate an inflammatory response in airway macrophages and respiratory epithelium leading to tissue damage that can result in respiratory illnesses in susceptible individuals.^7,27,29^ HAP is thought to be particularly damaging to lungs, as fine particulate matter (PM_2.5_) which is the by-product of incomplete combustion, penetrates deep into the alveoli of the lung.^30–32^

**Fig. 1:**
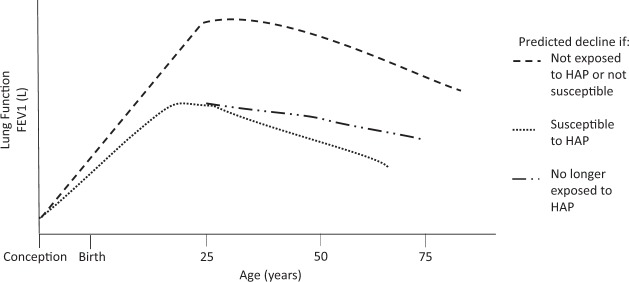
This hypothesized model shows the natural history of lung function, measured as % predicted forced expiratory value in one second (FEV1) for age. Predicted decline varies depending on the following scenarios (1) if the individual is not exposed to household air pollution (HAP) or not susceptible to respiratory illness, (2) exposed to HAP or susceptible, or (3) no longer being exposed to HAP. Those who are exposed or susceptible die from disease at a younger age, whereas those who cease exposure may reach disability but prolong life. This progression starts from conception, indicating that HAP exposure has a lifelong impact on lung function

In utero exposure to HAP may also affect lung development and lung function across the life span.^33^ However, data on the natural variability of lung function over a person’s lifetime are limited, and no long-term population-based studies have been conducted.^33^ The maximal attainment of lung function has been shown to be influenced by genetic pre-disposition, ambient air pollution, prematurity, and nutritional status.^34^ Those who may not reach their maximal lung function will have lower spirometric measurements throughout their lifetime.^34^ HAP exposure may affect maximal obtainment of lung function and subsequent lung function decline, as hypothesized in Fig. 1. Previous studies have explored the relationship between tobacco smoking and COPD in detail,^35^ and have theorized that some people are more susceptible to respiratory disease.^34^ The rate of lung function decline is heterogeneous as some people likely experience periods of rapid decline followed by slower decline.^34,35^ Similar mechanisms may apply in the case of HAP exposure, where noxious particles, such as particulate matter and carbon monoxide, may affect lung development starting in utero.^36^

Although biological plausibility and several observational studies support an association between HAP exposure and respiratory diseases, existing literature is either lacking or conflicting, limiting our ability to make causal inferences. There are few randomized controlled trials evaluating the effect of reducing HAP exposure on respiratory health outcomes. As a result, existing reviews and meta-analyses rely primarily on case-series and observational studies. The goal of this review is to summarize the systematic reviews and meta-analyses available for each respiratory disease then update this evidence a summary of available literature since the publication of these reviews.

## Results

Our primary search yielded 11 eligible systematic reviews, summarized in Table 1. The manuscripts from the secondary search, of which 19 were included in this paper, are summarized in Table 2. Based on our scoping review, HAP exposure may be associated with ALRI, COPD, tuberculosis, pneumoconiosis, head and neck cancer, and lung cancer. All of the systematic reviews included studies that were heterogeneous in methods and results. None of the systematic reviews had an objective measure of HAP exposure, instead exposure was often based on proxies and self-reporting. Furthermore, biomass fuel type was inconsistent between each study. Many of the studies do not separate cooking and heating and some include women only or both men and women.

**Table 1:** Qualitative overview of included systematic reviews

Authors (Year of publication)	Number of studies included	Meta-analysis (if yes, number of studies)	Exposure	Relevant outcome	Effect size	Bias & heterogeneity
Acute respiratory infections (ARI) and acute lower respiratory infections (ALRI)						
Po et al.^5^ (2011)	8	Yes (8)	Household combustion of wood, dung, crop residue, or charcoal indoors in non-industrialized or domestic settings	ARI and ALRI in children	ARIs in children exposed to biomass fuel smoke compared to those exposed to cleaner fuel (pooled OR = 3.52, 95% CI 1.94–6.43)	The Begg funnel plot asymmetry and the Egger test indicated publication bias after removing one outlier study. Significant heterogeneity was found among studies and so a random-effect model was used (*I*^2^ = 91.3%, *p* < 0.001)
Jary et al.^6^ (2016)	8	No	Air pollution from indoor burning of any solid fuels (wood, charcoal, animal dung, crop residues, and coal) for household purposes. This included studies that quantified exposure through direct measurement of specific pollutants, questionnaires regarding exposure history, comparison of groups exposed to types of exposure (e.g. different stove types), or before and after an exposure reduction intervention	ALRI in adults including pneumonia, acute bronchitis or bronchiolitis in adults. This included studies that defined the outcome as “acute” or specified duration of less than 14 days, even if infection was not confirmed, assuming that acute respiratory illnesses in the absence of underlying disease would likely be infectious in origin	Two of the studies documented increased risk of ALRI, two documented an unadjusted association, and the remaining four documented no association to ALRI and HAP	The Liverpool Quality Assessment Tool used indicated a “moderate/high” or “high” level of bias for case-control and cross-sectional studies. Risk of bias was moderate in at least 2 out of 4 domains for cohort studies. Meta-analysis was not possible due to methodological heterogeneity in exposure and outcome assessment
Misra et al.^7^ (2012)	24	Yes (9)	Use of solid and biomass fuels defined as (1) availability of measurements of HAP and/or exposure that demonstrate substantive exposure differential, (2) child carried while cooking, and (3) fuel use: unprocessed solid fuels compared to clean(er) fuels such as liquefied petroleum gas and electricity (fuels for comparison need to be specified)	At least one ALRI (pneumonia, emphysema, bronchiolitis, bronchiolitis) reported in children by a caregiver, study personnel or physician, death certificate or verbal autopsy, or detected in nasopharyngeal swab culture or nasopharyngeal aspirate immunoflouroscent microscopy	16 studies reported significant ORs (1.38–6.0) of ALRI exposed to HAP. Meta-analysis of 9 studies found that children exposed to HAP were more likely to have ALRI than those not exposed (pooled OR = 2.51, 95% CI 1.53–4.10)	Funnel plot and Egger’s test did not indicate presence of publication bias. There was heterogeneity among the studies (*I*^2^ = 93%) which led authors to use a random effect model
Jackson et al.^8^ (2013)	36	Yes (36)	Use of biomass fuels for cooking or a description of indoor smoke	Severe ALRI, defined differently depending on study setting: 1) hospital-based study: hospitalization for pneumonia or bronchiolitis in children under five years of age 2) community-based studies: presence of chest indrawing in a child with cough and difficulty breathing with increased respiratory rate for age within the WHO cut off for respiratory rate	HAP exposure increased risk of severe ALRI (pooled OR = 1.6, 95% CI 1.1–2.3)	Study indicated signs of recall bias, interviewer bias, and misclassification bias. Also found significant variation in confounder variation between studies. There was significant heterogeneity between studies (*I*^2^ = 82%)
Tuberculosis						
Kurmi et al.^9^ (2014)	12	Yes (12)	Smoke from solid fuel burning	TB defined by microbiological criteria (sputum smear alcohol-fast bacilli-positive) or doctor-diagnosed active TB	Positive association between solid fuel use and TB. Adjusted pooled effect for all types of solid fuel (OR = 1.43, 95% CI 1.07 to 1.91) was greater than for those using kerosene only (OR = 0.70, 95% CI 0.13 to 3.87) and mixed fuel (kerosene and biomass) (OR = 1.30, 95% CI 0.20 to 8.63)	The Egger plot indicated no publication bias (*p* = 0.14). Significant heterogeneity was found between studies (*I*^2^ = 70.8%, *p* < 0.001)
Lin et. al.^10^ (2014)	16	Yes (15)	Combustion of solid fuel (defined as coal/lignite, charcoal, wood, straw/ shrubs/ grass, animal dung or crop residues) for cooking and/or heating. Reference group included clean or non-solid fuels (electricity, liquefied petroleum gas, natural gas, biogas, and kerosene)	Latent tuberculous infection (LTBI) diagnosed by skin test or by (IGRA), active TB disease or TB mortality	No significant association between HAP exposure and TB. Case-control studies (Pooled OR = 1.17, 95% CI 0.83–1.65) and (Pooled OR = 1.62, 95% CI 0.89–2.93) for cross-sectional studies	8 of 10 case-control studies had high risk of bias for exposure assessment as recorded type of fuels used as a proxy to determine HAP exposure. Heterogeneity between case-control studies was substantial (*I*^2^ = 56.2%, *p* = 0.03. 4 of 6 cross-sectional studies had high risk for bias for outcome ascertainment as TB diagnosis was not based on bacteriological results or standardized criteria. Heterogeneity between cross-sectional studies was significant (*I*^2^ = 80.5%, *p* = 0.001). The Egger test and Begg test revealed possible risk of publication bias. No significant small study bias in funnel plot concluded
Asthma						
Po et al.^5^ (2011)	9	Yes (9)	Household combustion of wood, dung, crop residue, or charcoal indoors in non-industrialized or domestic settings for all age groups, gender, interventions, and study designs	Asthma in children and women	No significant association with HAP exposure and asthma. Children: (Pooled OR = 0.50, 95% CI 0.12–1.98); Adults: (Pooled OR = 1.34, 95% CI 0.93–1.93)	The Begg funnel plot asymmetry and the Egger test indicated publication bias after removing one outlier study. Both meta-analyses found significant heterogeneity among studies in children (*I*^2^ = 88.6%, *p* < 0.001) and in women (*I*^2^ = 58.6%, *p* < 0.05) and used random effects models
COPD						
Po et al.^5^ (2011)	12	Yes (12)	Household combustion of wood, dung, crop residue, or charcoal indoors in non-industrialized or domestic settings	COPD and chronic bronchitis in women	Exposure to biomass fuel smoke was significantly associated with COPD (OR = 2.40, 95% CI 1.47 to 3.93). Exposure to biomass fuel smoke was significantly associated with chronic bronchitis (OR = 2.52; 95% CI 1.88 to 3.38)	The Begg funnel plot asymmetry and the Egger test indicated publication bias after removing one outlier study. Chronic bronchitis: Borderline, nonsignificant heterogeneity among studies was found (*I*^2^ = 47.3% *p* = 0.09) but random effects models were still used. Egger test and funnel plot asymmetry suggested publication bias COPD: Heterogeneity was found among studies (*I*^2^ = 67.2%, *p* < 0.001) and random effects models were used
Kurmi et al.^11^ (2010)	23	Yes (23)	Domestic use of solid fuels	COPD was defined according to ATS and/or GOLD criteria, using the spirometry criteria of a forced expiratory volume in 1 s (FEV1)/forced vital capacity (FVC) ratio < 70% or physician diagnosis. Chronic bronchitis was defined according to Medical Research Council (MRC) criteria	Positive associations between the use of solid fuels and COPD (pooled OR = 2.80, 95% CI 1.85 to 4.0) and chronic bronchitis (pooled OR = 2.32, 95% CI 1.92 to 2.80)	The Begg funnel plot and Egger test did not show indications of publication bias. For both COPD and chronic bronchitis, heterogeneity between studies was found (lung function defined COPD: *I*^2^ = 91.8%, *p* < 0.001; physician defined COPD: *I*^2^ = 96.9%, *p* = 0.17; CB: *I*^2^ = 68.9% *p* < 0.001). There was heterogeneity in the pooled risk estimates for COPD and chronic bronchitis across studies, possibly due to variation in exposure between different settings and types of fuels and stoves used. Meta-regression found that COPD studies diagnosed on lung function criteria, year of publication and the year that study was conducted were significant contributors to heterogeneity. No heterogeneity from meta-regression were found for studies of chronic bronchitis
Pneumoconosis						
No systematic reviews have been published.^34^						
Head and neck cancers						
Josyula et al.^13^ (2015)	14	Yes (14)	HAP from all solid fuel types (coal, wood and mixed exposures) that were primarily derived from household cooking and/or heating and not from other forms of urban/outdoor air pollution or occupational exposures	Oral cancer, Pharyngeal cancer, Laryngeal cancer, Esophageal cancer, nasopharyngeal cancer	HAP was associated with oral (OR = 2.44; 95% CI 1.87–3.19); nasopharyngeal (OR = 1.80; 95% CI 1.42–2.29; pharyngeal (OR = 3.56; 95% CI 2.22–5.70) and laryngeal (OR = 2.35; 95% CI 1.72–3.21) cancers. The elevated risk for esophageal cancer (OR = 1.92; 95% CI 0.82–4.49) was non-significant	Funnel plot did not indicate publication bias. Heterogeneity was found for studies of nasopharyngeal cancer (*p* = 0.09). No significant heterogeneity was found for studies of oral (*p* = 0.93); pharyngeal (*p* = 0.99), esophageal (*p* = 0.53) and laryngeal (*p* = 0.49) cancers
Lung Cancer						
Kurmi et al.^14^ (2012)	28	Yes (28)	Biomass and solid fuel smoke, coal smoke	Lung cancer	Coal smoke had a slightly stronger association with lung cancer than biomass smoke but the confidence intervals overlap (Coal smoke: pooled OR 1.82, 95% CI 1.60–2.06; biomass smoke: pooled OR = 1.50, 95% CI 1.17–1.94). The risk of lung cancer from solid fuel use was greater in females (pooled OR = 1.81, 95% CI 1.54–2.12) compared to males (pooled OR = 1.16, 95% CI 0.79–1.69)	Begg funnel plot and Egger test indicated publication bias. There was significant heterogeneity across studies (*I*^2^ = 562.7%, *p* < 0.001). No significant heterogeneity was observed in the different strata for HAP exposure studies
Bruce et al.^16^ (2014)	14	Yes (14)	Biomass fuel exposure including wood, straw, grass, crop waste or residue, animal dung and charcoal	Lung cancer as cancer of any histological type emanating from the lung, trachea or bronchus	Association between biomass fuel use and lung cancer, when excluding studies without clean reference, was an OR 1.21 (95% CI 1.05–1.39) for men and 1.95 (95% CI 1.16–3.27) for women	Egger’s and Begg’s tests did not indicate publication bias but more than half the studies did not describe a reference category. Studies with men had no heterogeneity (*I*^2^ = 0%). Heterogeneity was higher in studies with women (*I*^2^ = 51%, *p* = 0.01) which may be explained by differences in exposure levels (i.e. developed vs developing settings) and aspects of methodology (i.e. type of fuel used in comparison group)

**Table 2:** Qualitative overview of included manuscripts published after systematic reviews

Authors/ Year of publication	Study design	Sample size	Study population	Exposure	Outcome	Adjustment for confounders	Effect size
Acute respiratory infections (ARI) and acute lower respiratory infections (ALRI)							
Bates et al.^43^ (2013)	Case-control	452 cases, 465 controls	Children 2–35 months old in Bhaktapur, Nepal. Cases: Children with ALRI or Severe ALRI; Controls: age-matched children without ALRI or Severe ALRI	Self-report on use of household cooking and heating appliances. Stoves and cooking fuels were confirmed by inspection	ALRI: cough or breathing difficulty combined with fast breathing ( > 50 breaths/min for children 2–11 months of age, > 40 breaths/min for children ≥ 12 months of age). Severe ALRI: cough or breathing difficulty accompanied by lower chest wall indrawing	Maternal education and occupation, having one or more family members who smoke indoors, and living in a single-family dwelling or shared home	Relative to use of electricity for cooking, ALRI was increased in association with any use of biomass stoves (OR = 1.9, 95% CI: 1.24, 2.98), kerosene stoves (OR = 1.87, 95% CI: 1.24, 2.83), and gas stoves (OR = 1.62; 95% CI: 1.05, 2.50)
Ramesh Bhat et al.^44^ (2012)	Case-control	101 cases, 101 controls	Cases: Children under 5 years of age admitted to Udupi District Hospital with ALRI; Controls: Healthy children under 5 years of age presenting for immunization	Self-report by child’s mother of cooking fuel type (LPG, wood, kerosene, dung, crop residues)	Acute lower respiratory tract infection as defined by the 1995 WHO definition	None described	Cooking fuel other than LPG was associated significantly with acute lower respiratory tract infection (94.1% vs 7.6%, OR = 26.3, 95% CI: 10.5–65.7; *p* < 0.0001)
Patel et al.^45^ (2013)	Cross-sectional survey	3 surveys were conducted across India. The 3 surveys are: NFHS-1 (1992–1993): 88562 households, 89777 women age 13–49 surveyed; NFHS-2 (1998–1999): 91196 households, 92300 women aged 15–49 surveyed, NFHS-3 (2005–2006): 109, 041 households, 124385 women aged 15–49 surveyed	Included participants from rural and urban India. Inclusion from each survey NFHS-1: 36121 households, NFHS-2: 32715 households, NFHS-3: 29509 households. Inclusion criteria: youngest two children under 36 months of age and ever-married women ages 13–49.	Self-reported fuel type: high polluting fuels (wood, agricultural waste, dung, straw), medium polluting fuels (coal/lignite, charcoal, kerosene), low polluting fuels (LPG, natural gas, electricity)	Acute lower respiratory tract infection was defined as cough with rapid breathing in the two-week period prior to the survey assessment	NFHS-3 adjusted for anyone present in the household who smoked	Odds ratios of ALRI by survey compared to low polluting fuels: Medium Polluting fuel: NFHS-1: OR = 1.39, 95% CI: 1.01–1.92, *p* < 0.05. NFHS-2: OR = 1.47 95% CI: 1.22–1.75, *p* < 0.001. NFHS-3: OR = 1.31, 95% CI: 0.92–1.88. High polluting fuels: NFHS-1: OR = 1.48, 95% CI: 1.08–2.03; *p* < 0.05. NFHS-2: OR = 1.54, 95% CI: 1.33–1.77; *p* < 0.01. NFHS-3: OR = 1.53 (95% CI: 1.21–1.93; *p* < 0.001)
Mortimer, K et al.^46^ (2017)	Cluster randomized controlled trial. Intervention: cleaner burning biomass-fueled cookstove; Control: open fire cooking.	10,543 children (5,297 in intervention and 5,246 in control group) from 8,626 households across 150 community-level clusters	Children under 5 years of age living in rural Malawi.	Stove use monitoring was performed in a 10% subsample of intervention households at baseline and 12 months of use	Pneumonia: defined according to WHO Integrated Management of Childhood Illness	District, baseline age of child, sex, distance to nearest health center, number of children < 5 years living in household, number of people in household who smoke regularly, other sources of fire or smoke (other than cooking) to which child was exposed on a daily (or almost daily) basis, socioeconomic status, number of previous pneumonia episodes, and vaccination status	The pneumonia incidence rate in the intervention group was 158. (95% CI: 14.89–16.63) per 100 child-years and in the control group 15.58 (95% CI: 14.7–16.5) per 100 child-years, with an intervention versus control incidence rate ratio of 1.01 (95% CI: 0.91–1.13; *p* = 0.80)
Smith, K. R. et al.^47^ (2011)	Randomized controlled trial. Intervention: locally developed chimney stove. Control: wood fire use for cooking.	265 children in intervention, 253 in control	Pregnant women or children < 4 months of age in households using an open fire for cooking in an enclosed kitchen, in the San Marcos region of the Guatemalan highlands	Personal 48 h carbon monoxide measurements obtained with diffusion tubes as indicators for wood smoke exposure	Physician-diagnosed pneumonia: not defined, stated as without use of a chest radiograph. Secondary outcomes: fieldworker-assessed pneumonia (all and severe) and seven other conditions of physician-diagnosed pneumonia	Not described	There were 124 physician-diagnosed pneumonia cases in intervention households and 139 in control households (Rate Ratio = 0.84, 95% CI: 0.63–1.13; *p* = 0.26)
Tuberculosis							
Rabbani et al.^52^ (2017)	Case-control	Total of 356 women (178 cases and 178 controls)	Large secondary care hospital in Pakistan. Cases: Non-smoking 20 to 65-year-old women with pulmonary TB; Controls: Age and area of residence matched women suffering from other diseases	Self- reported type of kitchen (ventilated vs non-ventilated), age at which cooking was started, average daily cooking time, current and past use of specific types of cooking fuels (biomass which included wood, crop residues and animal dung; or cleaner fuels which included natural gas and LPG)	New pulmonary TB cases diagnosed by physician through sputum smear for acid-fast bacilli or chest radiograph	Household monthly income and second hand tobacco smoke	Current users of biomass fuel were at higher risk of pulmonary TB (adjusted matched odds ratio [mOR] = 3.0, 95% CI = 1.1–4.9) compared with nonusers. In comparison with former biomass users (women not using biomass for > 10 years), recent biomass users (women who switched from biomass to non-biomass ≤ 10 years ago), and current (lifetime) users were at a higher risk in a dose-response manner (mOR = 2.8, 95% CI: 0.9–8.2 and mOR = 3.9, 95% CI: 1.4–10.7, respectively)
Jubulis et al.^54^ (2014)	Case-control	60 cases, 118 controls	Recruited from a Large tertiary care hospital in Pune, India. Cases: Children less than 5 years of age with confirmed/probable TB; Controls: Healthy children aged less than 5 years of age	Parent or guardian self- reported tobacco smoke exposure, primary cooking fuel used. No quantification of hours exposed	TB cases were defined according to the WHO and India’s Revised National Tuberculosis Control Program guidelines as confirmed or probable TB	Age, sex, school attendance, household TB exposure, household food insecurity and vitamin D deficiency	Exposure to IAP was independently associated with TB (OR = 2.67, 95% CI 1.02–6.97)
Asthma							
Oluwole et al. (2017)^60^	Cross-sectional survey	1,690 children	Children aged 6–21 years attending primary and secondary schools in Ibadan, Nigeria.	Child’s parent or guardians report of household cooking fuel type: biomass (cow dung/animal residue, firewood, charcoal) or no biomass (LPG, electricity)	Asthma symptoms were defined according to ISAAC definition	Age, sex, maternal level of education, tobacco exposure, indoor environmental characteristics, indoor pet exposure	Biomass fuel was associated with increased odds of asthma symptoms: adjusted odds ratios were 1.38 (95% CI: 1.05–1.80) for nocturnal cough, 1.26 (95% CI: 1.00–1.61) for current wheeze, and 1.33 (95% CI: 1.05–1.69) for report of any asthma-related symptoms
Oluwole et al. (2017)^61^	Cross-sectional survey	1,690 children	Children aged 6–21 years attending primary and secondary schools in Ibadan, Nigeria	Parent or guardian household cooking fuel type: biomass (cow dung/animal residue, firewood, charcoal) or no biomass (LPG, electricity)	Asthma symptoms were defined according to ISAAC definitions	Age, sex, maternal level of education, tobacco exposure, indoor environmental characteristics, indoor exposure to pets, BMI	In adjusted analyses, biomass fuel use was associated with increased odds of severe symptoms of asthma (OR = 2.37, 95% CI: 1.16–4.84), but not with possible asthma (OR = 1.22, 95% CI: 0.95–1.56)
Kumar et al.^62^ (2017)	Cross-sectional survey	204,568 individuals of all ages	Indian Human Development Survey II, a nation-wide survey conducted across India	Self- reported type of fuel used (clean only or other), cooking stove type. Quantification of use was not reported	Self-reported previous diagnosis of asthma or cough with shortness of breath	Sex, age, marital status, completed years of schooling, tobacco smoking, chewing tobacco, alcohol use, vegetarian, nutritional status, wealth quantile, religion, caste, place of residence	The odds of reporting asthma were higher for individuals living in households using unclean fuels (OR = 1.21, 95% CI 1.08–1.34)
Gonzalez-Garcia et al.^63^ (2015)	Cross-sectional survey	5,539 adults of all ages	Adults of both genders older than 40 years of age in urban areas of five Colombian cities	Self-reported history of using wood for cooking	Wheezing: affirmative answer to the question “Have you ever had two or more attacks of wheezes causing you to feel short of breath?” Asthma: wheezing definition plus a post-bronchodilator FEV1/FVC ratio less than 70% of predicted	City of residence, sex, BMI, education, respiratory disease before 16 years old, first-degree relative with asthma, occupational gases or fumes exposure, occupational dust or particles exposure	History of wood smoke exposure for cooking was associated with wheezing (OR = 1.24, 95% CI: 1.02–1.50, *p* = 0.033) but not with asthma (OR = 1.07, 95% CI: 0.87–1.32, *p* = 0.50)
Gaviola et al.^64^ (2016)	Cross-sectional	4,325 participants, of whom 2,953 had complete questionnaires and spirometry	Adults aged 35 years of age and older in four sites in Peru	Self-reported daily use of biomass fuels	Adults were defined as having asthma if they met any of the following three criteria: (1) physician-diagnosis of asthma, (2) self-reported wheezing attack in the last 12 months, or (3) use of asthma medications in the last 12 months.	Age, sex, height, living at high altitude, smoking, BMI, hypertension, family history of asthma, socioeconomic status, urbanization	Current daily exposure to biomass fuel smoke (OR = 1.18, 95% CI 0.70 to 1.91) was not associated with asthma
COPD							
Miele et al.^71^ (2016)	Cross-sectional	4,325 participants, of whom 2,947 met eligibility criteria based on completion of data	Adults aged 35 years and older in four sites in Peru	Self-reported daily use of biomass fuels.	Chronic bronchitis: having self-reported phlegm production (or both cough and phlegm production) for at least three months each year in two consecutive years. COPD: post-bronchodilator FEV 1 /FVC less than the lower limit of normal for a given age, sex, and height	Age, sex, hypertension, BMI, history of asthma and post-treatment TB, pack-years of smoking, wealth index, living in an urban setting, living at high altitude	Daily biomass fuel use was associated with chronic bronchitis (Prevalence Ratio = 2.00, 95% CI: 1.30–3.07, *p* < 0.01).
Amaral et al.^66^ (2018)	Cross-sectional	18,554 subjects across 25 international sites	Adults aged 40 years or older from low-, middle-, and high-income countries	Self- reported use of solid fuels was defined based on whether the participant had used an open fire with charcoal, coal, wood, crop residues, or dung as the primary means of cooking or heating the house or water for > 6 months in their lifetime. Self- reported exposure levels assessed	Airflow obstruction: a post-bronchodilator FEV1/FVC less than the lower limit of normal (LLN), based on reference equations for white individuals from the third U.S. National Health and Nutrition Examination Survey	Age, sex, BMI, pack-years of smoking, cumulative years of exposure to dust in the workplace	There was no association between airflow obstruction and use of solid fuels for cooking or heating (OR for men = 1.20, 95% CI 0.94–1.53; OR for women = 0.88, 95% CI: 0.67–1.15)
Siddharthan et al.^72^ (2018)	Cross-sectional	12,396 participants	Adults aged 35–95 in six countries in Latin America, Sub-Saharan Africa, and Southeast Asia	Household air pollution exposure was defined as self- reported use of biomass materials as the primary fuel source in the home	COPD: postbronchodilator FEV1/FVC z-score less than or equal to 21.64 SDs of the Global Lung Function Initiative mixed ethnic reference population	Age, sex, daily cigarette smoking, body mass index, post-treatment pulmonary tuberculosis, and secondary education	Participants with household air pollution exposure were 41% more likely to have COPD (95% CI: 1.18–1.68) than those without the exposure, and 13.5% (95% CI: 6.40–20.6%) of COPD prevalence may be caused by household air pollution exposure
Pneumoconosis							
Singh et al.^77^ (2015)	Case-control	30 cases, 53 controls	Cases: patients aged 20–85 years with acanthrosis on bronchoscopy recruited from SMS Hospital in Jaipur, India. Controls: patients matched according to age, gender and smoking habits, without black patches on bronchoscopy	Self-reported hours of biomass exposure	Acanthrosis: black pigmentation of the mucosal lining of the tracheobronchial tree on bronchoscopy	Not described	Biomass exposure for the cases was 35.13 ± 55.86 h in a year and for the controls was 28.2 ± 40.09 h in a year; this was not statistically significant (*p* > 0.05). ORs not reported
Pilaniya et al.^78^ (2017)	Case-control	60 female participants. Based on bronchoscopy findings, participants were divided into three groups: Group 1: patients with bronchial anthracofibrosis, Group 2: patients with only anthracotic pigmentation without narrowing/distortion, and Group 3: patients with a normal tracheobronchial tree	Newly referred “respiratory symptomatics” aged 40 years and above with history of exposure to biomass fuel smoke	Self-reported hours of biomass fuel smoke exposure, number of years of cooking, and exposure index (average number of hours of exposure per day multiplied by the number of years of cooking)	Bronchial anthracofibrosis: (1) long-standing history of biomass fuel smoke exposure, (2) on HRCT, the occurrence of multifocal narrowing of involved bronchus when present and (3) visual confirmation on fiberoptic bronchoscopy of (a) bluish-black mucosal pigmentation, along with (b) narrowed/distorted bronchus	No adjustment described.	Patients in Group 1 had significantly higher exposure index as compared to the other two groups (Group 1 vs Group 2: *p* = 0.0001; Group 1 vs Group 3: *p* = 0.0001; Group 2 vs Group 3: *p* = 0.27). ORs not reported
Sandoval et al.^79^ (1993)	Case-series	30 patients at an outpatient clinic in Mexico City, Mexico who lived in the countryside	Clinical, radiologic and electrocardiographic evidence of Pulmonary arterial hypertension and cor pulmonale, and the antecedent of at least 10 years of domestic wood-smoke exposure, non-smokers, no known lung disease	Self- reported mean exposure time	Anthracosis: intense dark blue staining of the bronchial mucosa by direct visualization during bronchoscopy; anthrancotic pigment deposition on open lung biopsy	No adjustment for confounders	14 of 22 patients who underwent bronchoscopy with direct visualization were found to have anthracosis. 5 out of 5 patients who underwent open lung biopsy had anthrancotic pigment deposition
Ozbay et al.^80^ (2001)	Case-series	30 consecutive patients	30 patients over 2 years who presented to clinic with (1) clinical and radiological diagnosis of COPD and/or interstitial lung disease, (2) antecedent long-standing domestic biomass exposure, (3) non-smokers (4) no chronic lung disease, (5) living in a rural area	Self- reported mean exposure to biomass fuels	Observations of the patients, findings on high resolution chest computerized tomography, arterial blood gases	No adjustment	Mean biomass exposure was 3.96 (2–10) hours per week for a mean of 37 ±10 years. PaO_2_ (mmHg) 54.4 ±11.4, PaCO_2_ 45 ±8.9, 76% had increased lung volumes or diffuse emphysema, 76% had reticulonodular pattern and/or thickening of interlobular septa, 40% had ground glass appearance, 30% had in a honeycombing-lobe or segment

### Acute respiratory infections

Acute respiratory infections include both upper respiratory infections (URI) and acute lower respiratory infections (ALRI). Upper respiratory infections are defined as infections of the upper respiratory structure of the aerodigestive tract, including diagnoses such as the common cold and sinusitis.^37^ ALRI is an acute infection of the lung from a viral or bacterial cause resulting in inflammation of the lung. ALRI is the leading cause of death in children under 5 years of age,^38^ and a frequent cause of hospitalization for adults in LMICs.^2^ Risk factors include low birth weight, malnutrition, low socioeconomic status, and smoking.^6,8,39^

Existing literature has not specifically investigated the link between URI and HAP exposure, instead considering URI in combination with all other acute respiratory infections. A 2011 meta-analysis from Po et al. found that, in eight studies of acute respiratory infection, children were 3.52 times more likely to develop acute respiratory infections when exposed to HAP than those exposed to cleaner fuel or kerosene (95% CI 1.93–6.43).^5^ Among adults, the evidence is less clear, and existing studies have included charcoal in the comparison group, which is not a clean fuel,^40^ or did not adjust for confounders.^41,42^

There is no clear consensus on the association between HAP exposure and ALRIs in adults. Jary et al., the only systematic review investigating this relationship, included eight eligible studies.^6^ Two of the studies documented an increased risk of ALRI, two documented an unadjusted association, and the remaining four documented no association. A meta-analysis was not performed as the studies were too heterogeneous in methods and results. Since its publication, no other studies have been published that further evaluate the relationship between HAP exposure and acute lower respiratory infections in adults.

The majority of studies investigating the association between HAP exposure and ALRI have focused on children under 5 years of age, as they are thought to be more susceptible to respiratory infections.^7^ HAP has been associated with increased risk of childhood ALRI. A systematic review by Misra et al. examined studies investigating the relationship between HAP exposure and ALRI in children under five years of age.^7^ Of 24 studies included for review, 16 reported significantly elevated OR, ranging from 1.38 to 6.0, of ALRI in those participants exposed to HAP. Nine studies were included in the meta-analysis and found that children exposed to HAP were 2.51 times more likely to have ALRI than children without exposure (95% CI 1.53–4.10). This review did not have clear criteria for ALRI and included a spectrum of severity.

Unlike previous reviews which focused on ALRI of any severity, a meta-analysis conducted by Jackson et al. aimed to identify risk factors specific to severe ALRI, defined as hospitalization for pneumonia or bronchiolitis, in children under five.^8^ In the pooled analysis of five studies in LMICs, the overall odds ratio was 1.6 (95% CI 1.1–2.3), indicating that exposure increased the risk of severe ALRI.^8^

**Fig. 2:**
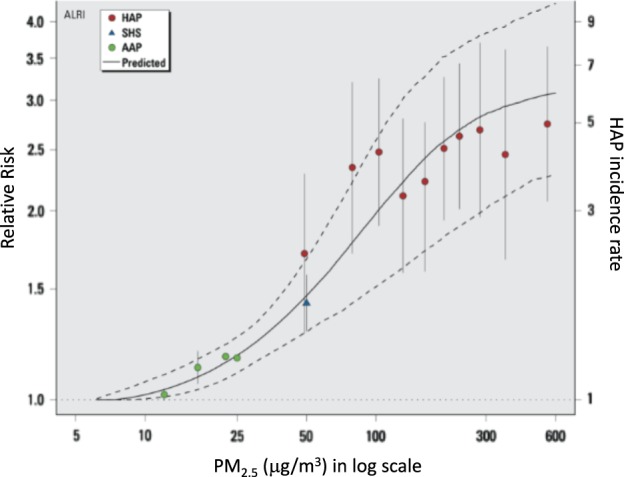
The exposure-response curves here, modified from Burnett et al. 2014, show the relationship between relative risk of ALRI in infants and particulate matter (PM_2.5_) exposure from household air pollution (HAP), second hand smoke (SHS), and ambient air pollution (AAP), with errors bars showing 95% confidence intervals. The solid line is the predicted values from the integrated exposure response (IER) model with dashed lines as the 95% confidence intervals. Figure was reproduced with permission from Burnett et al. 2014

Since these two systematic reviews, three studies^43–45^ found that HAP exposure was associated with higher chances of developing ALRI in children. In addition, one recent randomized controlled trial in Malawi documented no association between use of a cleaner burning biomass stove and decreased pneumonia in children.^46^ However, this trial may have suffered from insufficient reduction of exposure. A second randomized controlled trial in Guatemala (the RESPIRE study) demonstrated the importance of misclassification of exposure in understanding the relationship between childhood pneumonia and an intervention to reduce HAP exposure.^47^ In this study, the intention-to-treat analysis did not show a relationship between physician-diagnosed pneumonia and use of the improved biomass stove and chimney when compared with the control (OR = 0.84, 95% CI: 0.63–1.13) whereas the exposure-response analysis uncovered a significant relationship (OR = 0.82, 95% CI 0.70–0.98). This emphasizes the importance of obtaining personal exposure data, as not only do exposure-response analyses aid in the integration of exposure data, but they also help to understand the threshold at which reductions in HAP exposure lead to significant health benefits.^48^ As shown in Fig. 2, an exposure-response curve from Burnett et al., meaningful reductions in ALRI can only be achieved if PM_2.5_ concentrations are reduced to <35 µg/m^3^, the World Health Organization intermediate target goals for air quality in the household.^49^ This relationship may be applicable for all respiratory diseases discussed in this paper, but thus far has been most studied in childhood ALRI.

### Tuberculosis

Tuberculosis (TB) is a communicable infectious disease caused by the bacillus *Mycobacterium tuberculosis* and is spread by inhalation of the bacteria into the lungs. This is a disease primarily affecting those in LMICs, where 95% of TB deaths occur.^50^ Annually 250,000 children and 1.7 million adults die from TB.^50^ Risk factors include HIV, living in poverty, poor nutrition, and smoking.^26,50,51^

There is no clear consensus on whether there is a direct link between HAP exposure and TB in adults. Given the low incidence of TB disease in single-site studies, this association has been difficult to evaluate.^10^ Two systematic reviews from 2014, Kurmi et al.^9^ and Lin et al.,^10^ reached opposing conclusions on the association between TB and HAP exposure. Kurmi et al. identified 12 peer-reviewed studies that evaluated active TB, controlled for smoking and reported adjusted risk estimates.^9^ The adjusted pooled OR was 1.43 (95% CI: 1.07–1.91) for all 12 studies and 1.26 (95% CI: 0.95–1.68) when studies with physician-diagnosed TB were removed. This analysis concluded that an individual exposed to HAP has a 43% increased risk of having active TB compared to those using clean fuels. Lin et al. identified 15 studies that included adjusted risk estimates, of which 10 were case-control studies and 5 were cross-sectional studies.^10^ The pooled OR from case-control studies was 1.17 (95% CI: 0.83–1.65) and 1.62 (95% CI: 0.89–2.93) for the cross-sectional studies. This systematic review concluded that there was no strong evidence for a positive association between HAP exposure and TB. In fact, Lin et al. questions the conclusion drawn in Kurmi et al. since they calculated pooled OR using a fixed-effects model which may not be appropriate given the heterogeneity of the studies. Conversely, Lin et al. used the random-effects model to pool across heterogeneous studies.

Since these systematic reviews in 2014, few studies have been published evaluating the association between HAP exposure and TB. One case-control study among 178 women in rural Pakistan found a three-fold increase (OR: 3.0 95% CI: 1.1–4.9) in TB risk among current biomass fuel users compared to non-biomass users.^52^

There is also sparse literature evaluating the relationship between HAP exposure and TB in children. Only two studies could be identified that exclusively looked at HAP exposure and reported adjusted risk estimates: Ramachandran et al. and Jublis et al. yielded ORs of 6.9 (95% CI: 2.5–18.9)^53^ and 7.2 (95% CI: 1.4–44.5),^54^ respectively. Both of these studies suggest that HAP exposure increases the risk of TB in children. Since that review, there have not been any significant studies published evaluating the association between HAP exposure and TB. Future population-based studies are in progress but results have yet to be published.^55^

### Asthma

Asthma is a non-communicable respiratory disease that is caused by chronic inflammation of the airways and results in wheezing, chest tightness, and cough.^56^ Asthma may develop as an allergic disorder, and a large proportion of asthma cases have sensitization to aeroallergens.^57^ For the purpose of population-based studies, there is no clear definition of asthma, and studies use epidemiological definitions that include self-reported symptoms of wheezing in the past 12 months, physician reported wheezing or bronchodilator responsiveness.^56^ In 2015, approximately 400,000 people died of asthma worldwide, though asthma is considered severely under-diagnosed.^58^ Many risk factors are thought to be involved in the development of asthma, however, thus far smoking and occupational allergen exposure are the most clear risk factors.^59^ Interestingly, asthma is more prevalent in higher income countries and more urban areas.^56^

There is not a clear consensus on whether there is a direct link between HAP exposure and asthma in children or adults. Po et al. performed meta-analyses of four studies on asthma in children and five studies on asthma in adults, and did not find a significant association with HAP exposure (children: OR = 0.50, 95% CI 0.12–1.98; adults: OR = 1.34, 95% CI 0.93–1.93).^5^ Since the publication of that review, the studies published do not show conclusive results on the relationship between HAP exposure and asthma. Many are contradictory, with inconsistent settings and exposure definitions.^60–64^

### Chronic obstructive pulmonary disease (COPD)

Chronic obstructive pulmonary disease (COPD) is an adult disease characterized by irreversible airflow limitation due to a mixture of small airways disease and parenchymal destruction.^65^ The definition of COPD includes chronic bronchitis, defined by persistent daily phlegm for three months each year for at least 2 years,^66^ and emphysema, the destruction of the alveoli.^34^ In 2015, chronic obstructive pulmonary disease (COPD) caused 3.2 million deaths worldwide (95% CI 3.1–3.3 million).^67^ The World Health Organization ranks COPD as the fourth leading cause of mortality worldwide, 90% of which was in LMICs.^68^. Known risk factors of COPD include cigarette smoking, ambient pollution, genetics, poor socioeconomic status, and past history of TB.^34^

Exposure to HAP has been shown to be associated with COPD and this has been explored in multiple systematic reviews. After the definition of COPD recently changed to encompass chronic bronchitis and emphysema, prior studies looked at each disease separately.^34^ Two systematic reviews published months apart found that those exposed to HAP were more likely to develop chronic bronchitis and COPD.^5,11^ The most recent meta-analysis, by Smith et al., yielded a pooled OR of 1.94 for COPD (95% CI 1.62–2.33).^69^

Since these systematic reviews, several newer publications have investigated the association between COPD and HAP exposure. CRONICAS, a population-based study in Peru, documented that daily biomass fuel use for cooking was associated with COPD (prevalence ratio [PR] = 2.22, 95% CI 1.02–4.81)^70^ and chronic bronchitis (PR = 2.00, 95% CI 1.30–3.07).^71^ A recent publication, from the Burden of Obstructive Lung Disease Initiative (BOLD) investigators, questions previous literature as their analysis of post-bronchodilator spirometry measurements from 18,554 adults found no association between the use of solid fuels and airflow obstruction.^66^ These results, however, may be skewed since these data include high-income settings with little to no biomass fuel use. Another population-based study of 12,396 adults from 13 resource-poor settings documented that those with HAP exposure were 41% more likely to have COPD (OR = 1.41, 95% CI 1.18–1.68) than those without the exposure.^72^ This study is the first one to calculate population attributable risk factor and found that 13.5% (6.4%-20.6%) of COPD prevalence may due to HAP exposure.^72^

### Pneumoconiosis

Pneumoconiosis is an inflammatory lung disease that results in parenchymal scarring and nodularity and can eventually lead to fibrosis.^73^ Bronchial anthracofibrosis (BAF) is type of pneumoconiosis defined by black pigmented lesions along the bronchial mucosa with bronchial narrowing.^74^ This is diagnosed exclusively by bronchoscopic evaluation, therefore, limiting the diagnosis in LMICs as bronchoscopy is not widely available.^75^ Patients with bronchial anthracofibrosis suffer from dyspnea, cough, and hemoptysis.^76^ There are no systematic reviews or meta-analyses that evaluate the potential association between HAP exposure and bronchial anthracofibrosis. To attempt to shed light on this issue, Gupta et al. performed an extensive literature search to evaluate the association between HAP exposure and bronchial anthracofibrosis.^76^ From 17 studies and 6 case series, 1320 patients were identified with bronchoscopically confirmed BAF. The review suggested that HAP exposure might be a risk factor for bronchial anthracofibrosis, particularly in non-smoking women in rural areas.^76^ After that review’s publication, a 2015 case series study in India found that 30 consecutive participants exposed to HAP over a 13-month period were found to have black patches on their bronchial walls.^77^ They were matched with controls without black pigmentation. Compared to controls, cases were less likely to be exposed to HAP, although this was not statistically significant (OR = 0.57; 95% CI 0.19–1.74).^77^ Furthermore, a study in India in 2017 looked at 60 non-smoking females with respiratory symptoms and exposure to HAP. This study found that 40% of women with respiratory symptoms and exposure to HAP had bronchial anthracofibrosis diagnosed by imaging and fiberoptic bronchoscopy.^78^

While pneumoconioses such as bronchial anthracofibrosis can result in pulmonary fibrosis, there is no consensus that HAP exposure is associated with pneumoconiosis with higher risk of progression to fibrotic lung disease. Currently, no systematic reviews or meta-analyses have evaluated this potential association, but two case series exist. The first examined 30 Mexican rural women who had evidence of pulmonary hypertension and participants were exposed for an average of 59.1 years.^79^ Twenty-two patients underwent bronchoscopy and 14 had anthrancotic plaques present on visual examination. Transbronchial biopsy from 14 patients showed fibrosis. Pathology from open lung biopsies in 5 patients showed fibrosis with anthracotic deposits.^79^ In another case series of 30 women who were exposed to HAP over an average of 37 years and had a diagnosis of COPD received a high resolution computed tomography which consistently showed evidence of fibrosis. Two patients had open lung biopsies of which one had pathology showing end-stage fibrosis.^80^

### Head and neck cancer

Head and neck cancers encompass cancers of the lip, oral cavity, oropharynx, larynx, and nasopharynx and the associated structures in the regions of the head and neck. This group of malignancies is the ninth most common globally.^74^ In LMICs, this type of cancer is often caught in the late stages and has a high mortality rate.^81^ Tobacco consumption (smoked and smokeless), chewing areca nut, alcohol, and HPV infection have been associated with head and neck cancers.^74^.

Josyula et al. performed a meta-analysis investigating the relationship between HAP exposure and head and neck cancers.^13^ The results from three studies that adjusted for smoking indicated that HAP exposure is associated with a 2.56-fold increase in the risk of oral cancer (95% CI 1.80–3.64). Six studies yielded a pooled OR of 1.8 (95% CI 1.42–2.29) for nasopharyngeal cancer, although none of the individual studies controlled for smoking. Four studies reported smoking-adjusted OR of 3.56 (95% CI 2.22–5.70) for pharyngeal cancer. Five studies yielded smoking-adjusted OR of 2.35 (95% CI 1.72–3.21) for laryngeal cancer.^13^ Since this review, there have not been any newly published work disputing the association of HAP and head and neck cancers.

### Lung cancer

Lung cancer is the most common cause of cancer death worldwide^82^ with 1.59 million estimated deaths in 2012.^83^ Lung cancer is associated with smoking and more commonly found in high-income countries where smoking is prevalent. There is a rise in incidence of lung cancer in LMICs as tobacco smoking is increasing in popularity, particularly among men.^83^ Although lung cancer screening programs have been widely implemented in the US, they are less common in resource-poor settings because treatment options are not as widely available.^84^ Beyond smoking, known risk factors for lung cancer include environmental pollutants such as radon and asbestos, as well as chronic inflammation from pneumonia or TB.^84^

Lung cancer has been highly associated with HAP exposure in females. There is not a demonstrated association in males, likely due to reduced time spent cooking.^14,15^ In 2012, Kurmi *e*t al. performed a systematic review and meta-analysis of 28 studies evaluating HAP exposure on development of all types of lung cancer.^14^ The pooled analysis found a higher likelihood of developing lung cancer in women (OR = 1.81, 95% CI 1.54–2.12) but not in men (OR = 1.16, 95% CI: 0.79–1.69).^14^ This analysis controlled for tobacco smoking. Among fuel types, the fuel with the highest association with lung cancer was coal (OR = 1.82, 95% CI 1.60–2.06). The highest OR was among women in China who use coal for cooking. This meta-analysis may underestimate the impact of HAP on lung cancer risk as the studies selected did not have clean fuel controls. Subsequent to this meta-analysis, Bruce *et al*. found that among trials using clean fuels as a comparison group, the OR for lung cancer was 1.21 (95% CI 1.05–1.39) for men and 1.95 (95% CI 1.16–3.27) for women.^15^ There have not been any subsequently published manuscripts investigating this relationship that met our inclusion criteria.

## Discussion

Based on the literature summarized in this review, HAP exposure is associated with ALRI, COPD, tuberculosis, pneumoconiosis, head and neck cancer, and lung cancer. However, there has not been a causal link established between HAP exposure and respiratory disease. The Bradford-Hill criteria of causation allow for assessing causal evidence relating to environmental exposures and disease.^85^ An assessment of these criteria in the context of HAP and respiratory diseases is described in Table 3.^16^ Future studies should seek to strengthen consistency in outcome and exposure definitions, establish biological gradients through dose-response relationships, and strengthen experimental evidence through randomized controlled trials that implement interventions that adequately reduce HAP exposure.

**Table 3:** Assessment of Hill’s criteria of causation about the association between HAP exposure and respiratory disease

Criteria	Assessment
Strength of association	As outlined in this review, strong and significant associations have been documented between HAP exposure and ALRI, COPD, TB, pneumoconiosis, head and neck cancer, and lung cancer.
Consistency across populations	Consistency and reproducibility are lacking in the evidence presented in this paper due to heterogeneity between studies and inconsistent case and exposure definitions. Currently available studies are not easily amenable to meta-analysis due to lack of consistent definitions or diagnostic criteria for respiratory disease, instead relying on caregiver- or self-reported symptoms which lack diagnostic and etiological specificity. Exposure was also inconsistently defined and often not quantifiable.
Specificity	Since HAP exposure is linked to a wide range of respiratory diseases, specificity is no longer a widely accepted and used criteria.^91^
Temporality	Temporality has been shown through prospective cohort studies that have documented HAP exposure to precede respiratory diseases.^43,44,52,79^ There is still a need for randomized trials to lower PM_2.5_ to the World Health Organization standard (<35 µg/m^3^) and document if HAP reduction leads to an improvement in respiratory outcomes.
Biological Gradient (dose-response)	Many studies have failed to collect longitudinal exposure data to characterize the dose-response of HAP exposure to respiratory outcomes. However, evidence is available for a dose-response relationship between ALRI and HAP exposure. (Fig. 2)
Biological Plausibility	Strong evidence for biological plausibility exists linking noxious chemicals and particles in HAP to inflammation. Particulate matter, for example, has been hypothesized to stimulate an inflammatory response in airway macrophages and respiratory epithelium leading to tissue damage that can result in respiratory illnesses in susceptible individuals.^7,28,30,54,77,79,80^ HAP is thought to be particularly damaging to the lungs, as fine particulate matter (PM_2.5_) is a byproduct of incomplete combustion, penetrating deep into the alveoli.^31–33^
Coherence with natural history, animal studies	This scoping review has found evidence of higher risk of respiratory disease in LMICs where individuals have higher exposure to biomass smoke. Animal studies have also documented the harmful effects of HAP exposure.^92^
Experiment	Experimental or intervention-based epidemiologic evidence for HAP exposure and respiratory disease is thus far limited. Several studies and trials have been conducted with the goal to lower HAP by using more efficient biomass-burning cookstoves; however, it has become clear that reductions achievable by this approach fall short and fail to meet the World Health Organization intermediate target goals for air quality in the household (PM_2.5_ < 35 µg/m^3^).^49^ While it is intuitive that a switch to clean energy, such as liquefied petroleum gas (LPG), may prevent disease, there have been no published results from large-scale randomized controlled trials investigating this hypothesis.
Analogy	There is clear evidence from similar pollutants, such as cigarette smoke and outdoor air pollution

Clinicians should be aware of the increased risk of respiratory diseases and malignancies of the aerodigestive tract in patients who are actively being exposed to HAP or have been exposed at any point in their lives including in utero exposure. When evaluating respiratory symptoms of HAP exposed patients, clinicians should keep in mind that patients may not reach their maximal lung function^34^ if exposed early in life and may be more susceptible to the development of chronic respiratory diseases. As ALRI is one of the leading causes of death in children under 5 years of age,^1^ clinicians should be diligent in rapidly evaluating these children for pneumonia to provide antibiosis as quickly as possible. Although screening for all respiratory diseases and malignancies may not be possible in LMICs, when patients immigrate to developed countries clinicians need to be aware of this prior exposure and the effects on respiratory health when considering risk factors for implementing recommended screening guidelines. For example, although a patient may not have smoked cigarettes and would not qualify for lung cancer screening based on the current screening guidelines technically, biomass exposure was not considered specifically in these guidelines and may be substituted for smoking in calculating patient risk and need for screening.^86^ Better designed studies with a focus on characterizing exposure-disease relationship are needed to provide stronger recommendations.

Several studies and trials have been conducted with the goal to lower HAP by using more efficient biomass-burning cookstoves; however, it has become clear that achievable reductions fall short and fail to meet the World Health Organization intermediate target goals for air quality in the household (<35 µg/m^3^).^49^ As a result, scientists and policy-makers alike agree that more efficient biomass-burning cookstoves are unlikely to result in health benefits. While there is evidence for clean energy, such as liquefied petroleum gas (LPG), to prevent disease, there have been no published results from large-scale randomized controlled trials investigating this association. However, two ongoing LPG stove trials plan to fill this gap in the literature.^87,88^ If these trials can document health benefits associated with switching to LPG, further economic and implementation evaluations will be needed to understand if scaling up LPG interventions would be a valuable investment.

There are some limitations to our scoping review. First, time and manpower constraints limited our capacity to perform a full systematic review of original research articles as part of our secondary search. However, our primary goal was not to conduct a systematic review but instead to summarize current existing evidence for primary care physicians which we could accomplish with the approach presented here. Second, while we selected several chronic respiratory diseases to evaluate that have been linked to tobacco smoke exposure, we may have inadvertently not included some that may also be associated with HAP exposure. Several limitations arose based on the available literature included in this review. For instance, disease definitions vary greatly limiting comparability between studies. Additionally, HAP was inconsistently measured and was rarely quantified to show an exposure-response relationship. Many of the articles included did not consistently control for important confounders, such as tobacco smoke exposure in homes or outdoor air pollution levels. Lastly, there were varying levels of heterogeneity and publication bias among studies included in systematic reviews.

While there is a relationship between HAP exposure and many respiratory disease outcomes, better evidence in the form of randomized controlled trials reducing household air pollution are needed to strengthen this association. Further studies are needed to determine the best ways to screen for chronic respiratory diseases resulting from exposure to HAP, and identify adequate treatments, Moreover, clinicians should be aware that patients exposed to HAP may have a unique phenotype distinct from other environmental hazards such as tobacco smoke and occupational exposures.

## Methods

This scoping review described acute respiratory infections, tuberculosis, asthma, COPD, pneumoconiosis, head and neck cancers, and lung cancer, and how prevalence and burden of these diseases relate to HAP exposure. Respiratory diseases in this review were selected to encompass obstructive and restrictive lung disease and cancers attributed to tobacco smoke exposure. Although tobacco smoke is different than HAP cigarette smoke in chemical and particulate matter make-up, there are similarities between the two, it is a natural extension to study the same diseases for exposure to other smoke related exposures such as HAP.^89^

### Search strategy and study selection

We searched EMBASE, PubMed, and SCOPUS for systematic reviews and meta-analyses, reported the findings of these evaluations, and summarized the remaining literature since publication of each review. The search for systematic reviews was conducted by two informationists at the Johns Hopkins University Welch Library. We searched for common acute or chronic respiratory diseases (using the terms “acute respiratory disease” or “acute lower respiratory infection” or “pneumonia,” “tuberculosis” or “TB,” “asthma,” “chronic obstructive pulmonary disease,” “COPD”, “chronic bronchitis” or “emphysema,” “pneumoconiosis” or “pulmonary fibrosis,” “head and neck cancer,” “lung cancer”), each in combination with the terms “household air pollution,” “biomass,” or “indoor air pollution.” From the search terms provided to the informationists, a reference list of systematic reviews was provided to the authors. Selection of reviews for inclusion was undertaken by two authors (SS and DG). The literature search for systematic reviews occurred for reviews published before September 15, 2017. Based on these criteria, 63 systematic reviews were identified and 11 were included for this manuscript. We present Inclusion and exclusion criteria for systematic reviews in Table 4.

**Table 4:** Inclusion and exclusion criteria for systematic reviews

Inclusion	Exclusion
Exposure to household air pollution (HAP) caused by biomass fuels	Non-domestic exposures
Occurred in a low and middle- income country	English translation unavailable
Systematic review of the literature and/or meta-analysis	Non-peer reviewed sources
PRISMA standards met	
All ages	

The secondary literature search was performed for manuscripts after the publication of the chosen systematic review/s for each disease up until February 1, 2018. We searched PubMed and EMBASE for original research published subsequent to each of these reviews, using the same search terms as the primary search (“acute respiratory disease” or “acute lower respiratory infection” or “pneumonia,” “tuberculosis” or “TB,” “asthma,” “chronic obstructive pulmonary disease,” “COPD”, “ chronic bronchitis” or “emphysema,” “pneumoconiosis” or “pulmonary fibrosis,” “head and neck cancer,” “lung cancer”), each in combination with the terms “household air pollution,” “biomass,” or “indoor air pollution.” We searched each selected systematic review from the primary search in PubMed and reviewed each manuscript that cited each systematic review in PubMed Central. Hand searching was performed by examining the reference lists for relevant articles. Inclusion criteria for the secondary search of primary articles were similar: exposure to HAP caused by biomass fuels, all age, and conducted in a LMIC.

### Data abstraction and quality assessment

Each systematic review was evaluated by S.S. and D.G. and the most current systematic reviews that met our criteria were selected and mutually agreed upon by both authors. Abstracted statistics for each disease were confirmed by S.S., D.G., C.R., and M.C. All systematic reviews and meta-analyses included met Preferred Reporting Items of Systematic reviews and Meta-Analysis (PRISMA) standards.^90^ Each paper published subsequent to the last systematic review was evaluated by the authors and met the same criteria for inclusion as the systematic reviews.

## Data Availability

All included papers are published; no primary data are presented in this paper. As such, data sharing is not applicable to this article as no datasets were generated or analyzed during the current study.
